# Liberties of the genome: insertions of mitochondrial DNA fragments into nuclear genome

**DOI:** 10.18699/vjgb-24-53

**Published:** 2024-09

**Authors:** M.V. Golubenko, V.P. Puzyrev

**Affiliations:** Research Institute of Medical Genetics, Tomsk National Research Medical Center of the Russian Academy of Sciences, Tomsk, Russia; Research Institute of Medical Genetics, Tomsk National Research Medical Center of the Russian Academy of Sciences, Tomsk, Russia

**Keywords:** mitochondrial DNA, nuclear copies of mtDNA, NUMTS, genome evolution, mtDNA inheritance, митохондриальная ДНК, ядерные копии мтДНК, NUMTS, эволюция генома, наследование мтДНК

## Abstract

The transition of detached fragments of mitochondrial DNA into the nucleus and their integration into chromosomal DNA is a special kind of genetic variability that highlights the relation between the two genomes and their interaction in a eukaryotic cell. The human genome contains several hundreds of insertions of mtDNA fragments (NUMTS). This paper presents an overview of the current state of research in this area. To date, evidence has been obtained that the occurrence of new mtDNA insertions in the nuclear genome is a seldom but not exceptionally rare event. The integration of new mtDNA fragments into the nuclear genome occurs during double-strand DNA break repair through the non-homologous end joining mechanism. Along with evolutionarily stable “genetic fossils” that were integrated into the nuclear genome millions of years ago and are shared by many species, there are NUMTS that could be species-specific, polymorphic in a species, or “private”. Partial copies of mitochondrial DNA in the human nuclear genome can interfere with mtDNA during experimental studies of the mitochondrial genome, such as genotyping, heteroplasmy assessment, mtDNA methylation analysis, and mtDNA copy number estimation. In some cases, the insertion of multiple copies of the complete mitochondrial genome sequence may mimic paternal inheritance of mtDNA. The functional significance of NUMTS is poorly understood. For instance, they may be a source of variability for expression and splicing modulation. The role of NUMTS as a cause of hereditary diseases is negligible, since only a few cases of diseases caused by NUMTS have been described so far. In addition, NUMTS can serve as markers for evolutionary genetic studies. Of particular interest is the meaning of NUMTS in eukaryotic genome evolution. The constant flow of functionally inactive DNA sequences from mitochondria into the nucleus and its significance could be studied in view of the modern concepts of evolutionary theory suggesting non-adaptive complexity and the key role of stochastic processes in the formation of genomic structure.

## Abstract (ru)

Переход отдельных фрагментов митохондриальной ДНК в ядро и встраивание их в ДНК хромосом являются особым типом генетической изменчивости, характеризующим связь и взаимодействие двух геномов эукариотической клетки. В геноме человека содержится несколько сотен таких инсерций (NUMTS). Статья посвящена обзору современного состояния исследований в этой области. К настоящему времени получены данные о том, что появление новых инсерций мтДНК в ядерном геноме – редкое, но не исключительное событие. Встраивание новых фрагментов мтДНК в ядерный геном происходит при репарации двунитевых разрывов ДНК по механизму негомологичного соединения концов. Наряду с эволюционно стабильными «генетическими ископаемыми», встроившимися в ядерный геном миллионы лет назад и общими для многих видов и более крупных таксонов, существуют видоспецифичные, полиморфные и «приватные» NUMTS. Копии фрагментов митохондриальной ДНК в ядерном геноме человека могут интерферировать с митохондриальной ДНК при экспериментальных исследованиях митохондриального генома, таких как генотипирование и изучение гетероплазмии отдельных вариантов мтДНК, анализ метилирования мтДНК, определение числа копий мтДНК в клетке. Кроме того, в некоторых случаях инсерция нескольких копий полной последовательности митохондриального генома может имитировать наследование мтДНК от отца к детям. Вопрос о функциональной значимости NUMTS остается малоизученным. В частности, они могут являться источником изменчивости для модуляции экспрессии и сплайсинга. Роль NUMTS как причины развития моногенной наследственной патологии невелика, поскольку описано всего несколько случаев заболеваний, обусловленных NUMTS. Помимо этого, NUMTS могут служить маркерами для эволюционно-генетических исследований. Отдельный интерес представляет значение NUMTS в эволюции генома эукариот. Постоянный поток функционально неактивных последовательностей ДНК из митохондрий в ядро и его значение можно исследовать с точки зрения современных представлений теории эволюции, связанных с неадаптивностью сложности и центральной ролью стохастических процессов в формировании структуры геномов.

## Introduction

Mitochondrial DNA (mtDNA), which is situated outside the cell nucleus, is a special part of the genome. The establishment of symbiosis between the ancestor of the eukaryotic cell and the ancestor of the mitochondrion was the most important event in biological evolution, leading to the emergence of eukaryotes. During the further evolution of eukaryotes, most genes moved from mitochondria to the nucleus. This process apparently began immediately after the introduction of alphaproteobacteria into the cytoplasm of pro-eukaryotes (see review (Panov et al., 2020)). Moreover, it is assumed that the mosaic structure of eukaryotic genes is a consequence of the integration of DNA fragments from endosymbionts into the nuclear genome at the early stages of eukaryotic evolution, which, in turn, stimulated cell compartmentalization and isolation of the nucleus (Koonin, 2006; Rogozin et al., 2012).

Genomes of modern mitochondria contain a very limited set of genes. In most animals, mtDNA encodes only 13 subunits of respiratory chain proteins, ribosomal and transfer RNAs. The remaining genes have long and irreversibly “moved” into the nucleus. However, comparative genomic analysis shows that the integration of new mtDNA fragments into the nuclear genome continues, now being a microevolution process. So, in the chromosomal DNA of modern eukaryotes, including humans, there are many regions that are homologous to the mitochondrial genes. These sequences are called NUMTS – NUclear MiTochondrial Sequences. The placement of NUMTS in the genome is often associated with repetitive elements and transposons, but NUMTS themselves are not mobile genetic elements. The “mission” of NUMTS has not yet been revealed, but they are of interest both in a practical sense, because they may have a pathogenic effect, and in a theoretical aspect, since they may represent a different path of genome evolution.

The article is devoted to an overview of the current state of research on the NUMTS phenomenon and its role in the life of the human genome

## Prevalence of NUMTS in the human genome

Soon after the sequence of human mitochondrial DNA was determined, DNA fragments embedded in nuclear chromosomes and homologous to mtDNA were discovered (Tsuzuki et al., 1983). As the human genome was sequenced, the analysis of homology between NUMTS and modern mtDNA in humans and other species showed that the insertion of mtDNA fragments into chromosomes is an ongoing process (Mourier et al., 2001). NUMTS are found on all human chromosomes and are situated mostly in regions rich in various repeats. Development of new sequencing technologies, improvements in bioinformatics, and the accumulation of data on individual genomes lead to the identification of more and more such insertions, and it is becoming evident that NUMTS is a common phenomenon. The human reference genomic sequence GRCh37/ hg19 contains 766 insertions of mitochondrial genome fragments homologous to the modern human mtDNA reference sequence (Calabrese et al., 2012). Subsequently, analysis of data from the 1,000 Genomes Project (999 individuals from 20 populations) identified 141 polymorphic NUMTS sites in the nuclear genome, in addition to those insertions that are “fixed” in the human population. Of these, 42 % of polymorphic NUMTS were located in introns, 43 % were located in intergenic regions, and most of these NUMTS were “younger” than a million years old (Dayama et al., 2014).

A recent analysis of the complete genomes of 66,000 individuals, including more than 10,000 trios (Wei et al., 2022), has already identified more than 1,500 new NUMTS, the vast majority of which were rare in the population or “private”, i. e. found in only one individual. So, the incidence of de novo NUMTS insertions has been estimated to be approximately 1 in 10,000 births and approximately 1 in 1,000 tumors. Moreover, estimates of the time of integration into the nuclear genome obtained for several hundred NUMTS showed that 90 % of these events occurred no more than 100 thousand years ago (Wei et al., 2022). Some figures characterizing the diversity of NUMTS in the human genome are presented in Table 1. It is worth noting that the total length of NUMTS is about 630,000 bp (Tao et al., 2023), or approximately 0.02 % of the total length of the human genome.

**Table 1.**
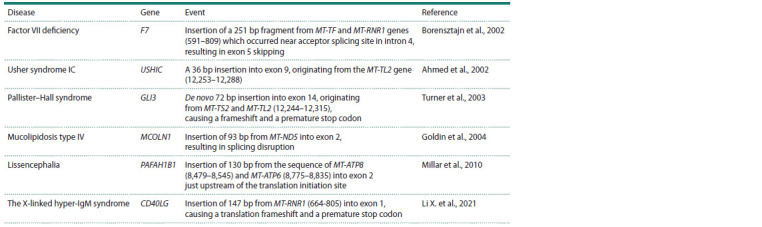
Characteristics of the NUMTS “landscape” in the human nuclear genome Notе. ND – no data.

Depending on the search and aligning algorithms, the minimal length of detected mtDNA fragments starts from 30 bp, and most of them are shorter than 500 bp. However, insertions of almost the entire mitochondrial genome sequence also occur. In particular, in the intergenic region on chromosome 4, there is an insertion 14,836 bp in length, homologous to a 14,904 bp region in the mtDNA sequence (positions 661–15,564) (Calabrese et al., 2012).

Mitochondria are not the only organelles that “send” fragments of their DNA to the nucleus. To the same extent, this process is characteristic of plastids (Zhang et al., 2024). In addition, this phenomenon may be more or less prevalent depending on the species. For example, the search for NUMTS in the genomes of 13 different species revealed large interspecific differences: the nematode, some dipterans (Anopheles, Drosophila) and puffer fish have only a few mtDNA fragments in their nuclear genome, while humans, some insects, and plants have several hundred NUMTS (Richly, Leister, 2004; Leister, 2005). Moreover, the number of NUMTS may depend on the genome size and speciation characteristics

These data suggest that the integration of mtDNA fragments into chromosomal DNA is not a rare event but a natural property of the human genome dynamics, and therefore it must be taken into account and should be explored.

## The mechanism for the emergence of new NUMTS

Almost all studies show that the general mechanism for the integration of mtDNA fragments into the nuclear genome is non-homologous end joining (NHEJ) as a way to repair double-stranded DNA breaks (Gaziev, Shaikhaev, 2010). Usually, NUMTS are associated with mobile genetic elements: for example, a study of 271 human NUMTS showed that most of them are located within 150 bp from repetitive elements (predominantly LINE and Alu repeats) or even within these sequences (Mishmar et al., 2004). A recent search and analysis of NUMTS in the genomes of 45 mammalian species has essentially confirmed this fact (Uvizl et al., 2024).

In a study from Japan (Onozawa et al., 2015), it was shown that DNA insertions belonging to the second class of “templated sequence insertion polymorphism” (TSIP) had some characteristics consistent with their occurrence as a result of double-strand breaks repair event with use of the mechanism of non-homologous end joining, and it is noteworthy that in more than 20 % of TSIP cases, mitochondrial DNA served as the “donor” DNA for such insertions (Onozawa et al., 2015).

According to the results of experiments on irradiation of chicken eggs, new insertions of mtDNA fragments were identified in 25 % of surviving embryos (2 out of 8) (Abdullaev et al., 2013). In the paper on the case of a pathogenic de novo NUMTS insertion leading to the development of Pallister– Hall syndrome, the authors note that the family where the affected child was born lived in an area exposed to the Chernobyl accident in 1986 (Turner et al., 2003). It is fair to assume that since ionizing radiation leads to double-strand breaks in DNA and the appearance of new NUMTS is associated with the process of repairing this damage, the probability of integration of mtDNA fragments into the nuclear genome increases after irradiation

It should be noted that in a non-dividing cell, the nuclear and mitochondrial genomes are separated from each other by a total of four membranes (the nuclear double membrane and the mitochondrial double membrane). To integrate a fragment of mtDNA into the DNA of a chromosome, this fragment should be able to enter the nucleus. To date, several possible ways of such transfer have been proposed. The most acceptable hypothesis is the assumption that mtDNA fragments that appear due to the impact of reactive oxygen species enter the cytoplasm as a result of changes in the mitochondrial membrane (opening of pores, mitochondrial fusion/fission, etc.), and then are transported into the nucleus using vacuoles (Puertas, González-Sánchez, 2020).

## NUMTS studies in evolutionary genetics

Depending on the time of their origin, NUMTS can provide information about the evolutionary history of the human species (Hazkani-Kovo, 2009). Two features of the evolution of NUMTS can be distinguished in comparison with the homologous mtDNA regions: firstly, NUMTS are pseudogenes, therefore selection does not affect them, and the mutation process is more “uniform”, and secondly, the rate of molecular evolution declines after integration into the nuclear genome, consistent with general differences in mutation rates between nuclear and mitochondrial DNA. That is, on the one hand, the “biological clock” for NUMTS works more precisely, and on the other hand, they are a kind of “genetic fossil” containing information about mtDNA haplotypes that might not have been preserved in modern populations, so they can serve as an “outgroup” for intraspecific phylogeny (Bravi et al., 2006). For example, two NUMTS in the human genome that are homologous to the CO1 gene contain nucleotide substitutions (compared to the reference mtDNA sequence) characteristic of the most ancient mitochondrial superhaplogroup L (Mishmar et al., 2004).

Using a comparative analysis of polymorphic NUMTS in the genomes of Homo sapiens sapiens, H. sapiens neanderthalensis and H. sapiens denisova, five insertions of mtDNA fragments were identified. These insertions occurred during the evolution of the genus Homo and have been preserved in the genomes of modern humans. Of these, two NUMTS originated from the mitochondrial genome of Denisovans and entered the modern human genome as a part of nuclear DNA. They were identified in the genomes of several Indonesians (Bücking et al., 2019). Analysis of NUMTS in the genomes of great apes revealed several fragments, for which the divergence of their sequence from modern mtDNA of these species indicated that they also entered the genomes of hominids as part of nuclear DNA due to admixture of unknown extinct species (Popadin et al., 2022). Interestingly, an analysis of the time of appearance of Homo-specific NUMTS in the human genome showed that the occurrence of a significant number (one third of the 18 analyzed) of insertions coincided in time with the estimated time interval of the origin of the genus Homo, as well as with drastic climate change, i. e. about 2.5–2.9 million years ago. Thus, speciation appears to be associated with an increase in the rate of insertion of new NUMTS into the genome. However, the question remains open whether these insertions are just markers of a period of genomic instability in the species’ history (“riders”) or whether they play a significant role in speciation, changing the structural and expression architecture of the genome (“drivers”) (Gunbin et al., 2017). The first hypothesis is supported by data on a similar “explosion” in the frequency of NUMTS in marsupial martens, which occurred during the same period (Hazkani-Covo, 2022). The second hypothesis deserves attention due to the fact that NUMTs are often found in regions of open chromatin associated with DNase I hypersensitivity and expression regulation (Wang, Timmis, 2013). The uneven rate of organelle DNA insertions into chromosomes during evolution is also demonstrated by homology analysis of NUMTS and the “parental” organelle genomes: the distribution of NUMTS by extent of their sequence identity to the mitochondrial genome shows that although these insertion events occur throughout the species history, the rate of the process is not constant. For example, in Homo sapiens, most NUMTS have 70 to 85 % identity with the mitochondrial genome, while in Phytophthora, the sequence identity is about 100 % (Hazkani-Covo, Martin, 2017).

## Pathogenic effects of NUMTS

Random insertion of any DNA fragment into exonic or regulatory regions of genes can have a pathogenic impact. Cases of hereditary diseases caused by de novo insertions of mtDNA fragments into nuclear genes have indeed been described, but it should be noted that they are rare (Table 2).

**Table 2.**
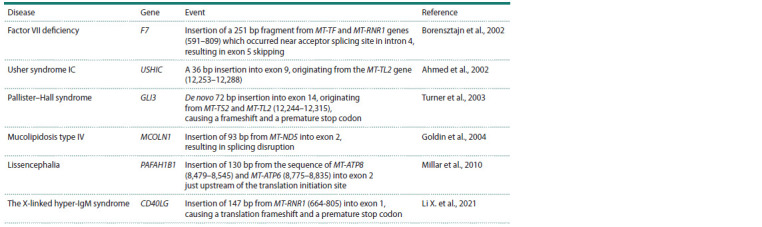
Known cases of diseases caused by insertions of mtDNA fragments

The first case of a disease associated with NUMTS was described in 2002. Severe coagulation factor VII deficiency was found in a patient who was a compound heterozygote: one copy of the gene had a 7 bp deletion, and the other had a 251 bp insertion from the MT-RNR1 gene into the polypyrimidine tract near the splice acceptor site in intron 4 of F7 (Borensztajn et al., 2002). In 2003, a sporadic case of Pallister–Hall syndrome was characterized: a de novo 72-bp insertion from mtDNA into exon 14 of the GLI3 gene resulted in a frameshift and the formation of a premature stop codon (Turner et al., 2003). Notably, the allele with this de novo NUMTS was of paternal origin. In addition, several other cases of pathogenic NUMTS disrupting splice sites or causing frameshifts have been published. Given the large number of genetic tests being performed (targeted and exome sequencing) that can potentially detect such insertions, we can conclude that pathogenic NUMTS in humans are extremely rare.

In contrast to the few cases of NUMTS leading to hereditary diseases and syndromes, de novo insertions within exons and regulatory sequences in malignant tumors are not so rare. In one study, 220 somatic “tumor-specific” NUMTS were identified within genes, and out of these, 13 were located in the coding regions of genes (including 3 and 4 that disrupted stop and start codons, respectively), and 16 were located in the 3′ or in 5′ untranslated regions (Wei et al., 2022). Possibly, accumulation of somatic NUMTS with time may also contribute to aging.

Recently, it was shown that insertions of mtDNA fragments into introns can affect gene expression, i. e. transcription and splicing, especially if the inserted fragments contain tRNA genes that are capable of forming secondary structures. In particular, one study examined the effect of such mitochondrial tRNA (nimtRNA) gene insertions on splicing, using a splicing reporter gene construct (Hoser et al., 2020). The experiments showed that nimtRNAs inserted into the intron of the reporter gene enhance pre-mRNA splicing, depending on their number and location, as well as the efficiency of splice site recognition, while the insertion of nuclear tRNAs did not have such an effect. In addition, this work demonstrated that partial deletion of nimtRNA(Lys), located in intron 28 of the PPFIBP1 gene, reduces the likelihood of inclusion of exon 29 in the mRNA (Hoser et al., 2020). Thus, some NUMTS may have a regulatory impact.

## NUMTS as a source of artifacts in mitochondrial DNA studies

MtDNA heteroplasmy

When studying mtDNA heteroplasmy, NUMTS can significantly interfere with the results, especially in the case of low levels of the mutant allele (Maude et al., 2019; Xue et al., 2023). In particular, G. Dayama et al. (2014) identified 59 po-sitions in mtDNA where false heteroplasmy caused by polymorphic insertions in the nuclear genome can be systematically detected. A comparison of enrichment methods for NGS (hybridization or long-range PCR) and alignment approaches (aligning reads on the whole genome or only on mtDNA, using different threshold levels for heteroplasmy detection) showed that a significant part of the “alternative” alleles in heteroplasmic positions actually correspond to NUMTS alleles, and this effect is more pronounced when using a low heteroplasmy threshold, a hybridization enrichment method, and mtDNA as the only reference for alignment. On the other hand, taking these factors into account leads to a decrease in coverage depth and to the omission of truly heteroplasmic positions in mtDNA (Li M. et al., 2012).

For the sample of a thousand individuals from the Swedish population, analysis of complete mitochondrial genomes showed that with an average mtDNA read depth of more than 2000x, about 40 % (373 out of 934) of mtDNA haplotypes have “heteroplasmic” positions with a variant fraction of more than 2 % (i. e. above the “noise level”) that is driven by variants in NUMTS (Sturk-Andreaggi et al., 2023). At the same time, 31 “heteroplasmic” positions were characterized by a proportion of the alternative (associated with NUMTS) allele of more than 10 %, but the authors note that in these cases the mtDNA reading depth was less than 100x (Sturk-Andreaggi et al., 2023). Given that mtDNA mutations leading to the development of mitochondrial diseases are also heteroplasmic, and the level of heteroplasmy can vary depending on the tissue, it is important to take NUMTS existence into account when performing genetic diagnostics (Yao et al., 2008).

## NUMTS and assessment of mitochondrial DNA methylation level

Studies of the mitochondrial DNA epigenetics produce contradictory results: some groups of researchers reveal a fairly high level of cytosine methylation in mtDNA, while others reveal a very low methylation level (see reviews (Byun et al., 2013; Hong et al., 2013; Zinovkina and Zinovkin, 2015; Maresca et al., 2015; Patil et al., 2019)). Analysis of these publications suggests that the resulting estimates of the proportion of methylated cytosines depend on the detection methods. Since NUMTS are essentially pseudogenes, they are expected to be methylated, and this is indeed supported by direct determination of methylation levels using Oxford NanoPore technology (Wei et al., 2022). In particular, our own studies showed an extremely low (at the scale of error rate) level of cytosine methylation in the regulatory region (D-loop) of mtDNA; this estimate was obtained by sequencing (NGS) of PCR products using sodium bisulfite-treated DNA as a template (Golubenko et al., 2018).

Recent publications have shown that the true level of cytosine methylation in mtDNA is indeed less than 1 %, and higher values are caused by “interference” of signals from nuclear pseudogenes (i. e. NUMTS) or the influence of nucleotide context on base calling (Bicci et al., 2021; Guitton et al., 2022; Shao et al., 2023). However, it should be noted that DNA methyltransferase DNMT1 was in fact found in mitochondria, and its mitochondrial isoform is synthesized from an alternative promoter (Shock et al., 2011). So, the existence of DNA methylation in mitochondria cannot be completely excluded, and for instance, it might occur during programmed or pathological deactivation/degradation of mtDNA.

## NUMTS and mtDNA copy number estimation

The presence of NUMTS is a major difficulty in the development and use of methods for quantifying mtDNA copy number per cell, i. e. the ratio of the number of copies of a mtDNA region to the number of copies of a “control” nuclear gene. Currently, several methods are used to determine mtDNA copy number, the most popular of them is real-time PCR using fluorescent dyes, including TaqMan probes, as well as digital PCR. Designing primers that could anneal only to mtDNA involves significant difficulties, since almost the entire mtDNA sequence is represented in the nuclear genome, and moreover, a significant part of it is represented by a large number of fragments, sometimes comparable to the number of mtDNA copies in the cell. In addition, each individual lacks on average four NUMTS from the reference genome sequence (Wei et al., 2022). Thus, even a thorough BLAST analysis for the primers and probes sequences that takes into account sequence identity level of NUMTS and mtDNA, adding the high level of polymorphism of mtDNA itself, does not always allow to make an adequate assessment of the mtDNA copy number in a cell. Probably, several regions of mtDNA should be used simultaneously for these purposes

## NUMTS and forensic studies

Considering that events of de novo insertion of mtDNA fragments into the nuclear genome are not extremely rare and their length can be large, data obtained by forensic experts during molecular genetic examinations should be interpreted with caution. If a large mtDNA insertion persists in the genome for several generations, or if a child “inherits” part of the parent’s mtDNA genotype in its nuclear genome due to a de novo insertion, then analysis of a total DNA sample will yield a mixture of the two haplotypes (see, for example, Lutz- Bonengel et al., 2021) and could potentially lead to false DNA identification. In addition, co-amplification of NUMTS can probably occur in other cases (for example, when analyzing a degraded DNA sample, where the copy number of mtDNA is low and comparable to the copy number of homologous NUMTS in the sample under study (Bravi et al., 2006)). It was shown that when analyzing data obtained using multiple parallel sequencing (NGS, or MPS) methods, it is possible to filter out NUMTS using bioinformatics methods, but in forensic studies, researchers often deal with degraded DNA samples from which only short fragments can be obtained, and in this case bioinformatic “filtering” is less effective (Marshall, Parson, 2021).

“Paternal inheritance” of mtDNA

The history of the search for the possibility of paternal inheritance of human mtDNA is quite interesting. Reports on cases with a supposed contribution of mtDNA from sperm mitochondria to the general pool of mtDNA in the zygote and developing organism keep appearing in the scientific press. A recent sensational publication on this topic (Luo et al., 2018) demonstrated three pedigrees where children inherited their father’s mtDNA in a certain proportion and then passed it to some of their children in the same proportion. The authors suggested that the possibility of paternal mtDNA inheritance was due to a variant of a nuclear gene with a dominant effect. This paper gave rise to an extensive scientific debate in the following publications (Luo et al., 2019; Lutz-Bonengel, Parson, 2019; McWilliams, Suomalainen, 2019), and also stimulated further research in this area, which showed that these cases can be explained by insertions of concatemers (tandem linear copies) of mtDNA into the nuclear genome, representing the so-called mega-NUMTS (Wei et al., 2020; Bai et al., 2021). For example, one concatemer identified on chromosome 14 consisted of 50 copies of mtDNA (Lutz-Bonengel et al., 2021).

And yet, the final verdict on the topic of “intergenerational transmission of the paternal mitochondrial genome” should not be rendered, since it is still unclear how exactly the obligate elimination of paternal mtDNA is ensured in the zygote. Some studies show that there is no universal mechanism for such elimination. For example, in nematodes, sperm mitochondria are “digested” in the zygote after fertilization using the autophagy mechanism, but if it does not happen, then the embryos are not viable. In mice (and, probably, humans), the paternal mitochondrial genome is eliminated already in the mitochondria of the sperm, which, therefore, do not contain mtDNA at all. However, if for some reason mtDNA is not completely degraded, then its presence in the mouse embryo can be traced up to the morula stage (Luo et al., 2013)

at the Institute of Experimental Medicine in St. Petersburg, attract attention in this regard. In some embryos and newborn mice, human mtDNA was retained in some tissues, and in some cases, it was transmitted to F1 and even F2 offspring (Sokolova et al., 2004; Bass et al., 2006). Later it was demonstrated that mouse and human mitochondria successfully merged with each other in cell fusion experiments, and also produced “xenocybrids” containing the mouse cell nucleus and human mitochondria, although they could not grow in a medium requiring normal mitochondrial function (Yoon et al., 2007). Thus, the formation of chimeric human and mouse mitochondria is possible, and it is likely that after microinjection into the mouse zygote, human mitochondria were combined with mouse ones. It is unknown whether human mtDNA was integrated into mice nuclear genome in this case, and additional experiments are needed to clarify this, but given that human mtDNA was found in much less than half of the offspring and not in all tissues, and that injections of mitochondria were carried out into already fertilized zygotes, it can be assumed that it was not contained in the nucleus but specifically in the mitochondria.

## Conclusion

The phenomenon of translocation of mtDNA fragments into the nuclear genome is a special type of genomic variability that deserves close attention. In recent years, it has been shown that the prevalence of these events is much higher than previously thought. The mitochondrial genome unexpectedly appeared not as a subordinate “prisoner” of the eukaryotic cell, but as an independent source of new material for the nuclear genome. The role of this phenomenon in the life of the cell remains unknown. Perhaps its understanding goes beyond the framework of classical “deterministic” genetics and can be explored in the paradigm of a new “postmodern” approach, which assumes the multiplicity of patterns and processes of evolution for the living forms, as well as the central role of unpredictable events, that is, the non-adaptiveness of the main path of evolution (Koonin, 2014). This suggests the need for stochastic transformation of the genome in evolution, “genomic instability” (Khesin, 1985) or “genome liberties” (Puzyrev, 2002). It is worth noting that while genetics as a science was developing in the frame of classical simplified concepts regarding genes, mutations and heredity, the ideas of gene mobility, as well as abruptness of mutational changes and multiplicity of gene manifestations at the phenotypic level, were expressed by many researchers since the end of the 19th century (see in: Puzyrev, 2002; Golubovsky, 2011).

It is interesting that in the model of the evolution of entropy and genome complexity proposed by E.V. Koonin, two scenarios are considered, including a “high-entropy” way, which is accompanied by a decrease in gene density, and the opposite “low-entropy” way, which consists of genome optimization and maximum information density (Koonin, 2014). We can say that the transfer of mtDNA fragments into the nuclear genome contributes to its evolution in the “high-entropy” mode, while the mitochondrial genome itself followed the opposite “low-entropy” scenario. It is noteworthy that these two paths are governed, among other things, by the effective population size, which is small in the first case (“high-entropy”) and large in the second (“low-entropy”); this rule surprisingly corresponds to the diploidy (in most cases) of the eukaryotic nuclear genome, on the one hand, and the large number of mitochondria inhabiting them, on the other hand. It should also be noted that it is the simplification of the genome following an abrupt increase in its complexity that is assumed by this model as a general trend in evolution (Wolf, Koonin, 2013), and “an increase in the entropy of the genome ... can be considered as a “genomic syndrome”, as the inability of organisms with a small effective population size to cope with the spreading of selfish elements and other processes leading to the increase in entropy” (Koonin, 2014).

If we consider NUMTS from a “practical” point of view, it has now been demonstrated that nuclear copies of mitochondrial DNA fragments in the human genome can introduce some noise into the data obtained from experimental studies of the mitochondrial genome, but may also carry some functional load. At least, they serve as a variability source for modulation of expression and splicing. In addition, they have significant potential as polymorphic markers for evolutionary genetic studies. Also, NUMTS may be involved in speciation, but this issue requires further research. The significance of NUMTS in the development of monogenic hereditary pathology is apparently small, and their role in aging and the development of multifactorial diseases, including cancer, remains to be studied.

## Conflict of interest

The authors declare no conflict of interest.
